# Comparison of Models for Tumor Recurrence after Liver Transplantation for the Patients with Hepatocellular Carcinoma: A Multicenter Long-Term Follow-Up Study

**DOI:** 10.3390/cancers11091295

**Published:** 2019-09-03

**Authors:** Young Chang, Yuri Cho, Jeong-Hoon Lee, Yun Bin Lee, Eun Ju Cho, Su Jong Yu, Dong Hyun Sinn, Bo Hyun Kim, Seoung Hoon Kim, Nam-Joon Yi, Kwang-Woong Lee, Jong Man Kim, Joong-Won Park, Yoon Jun Kim, Jung-Hwan Yoon, Jae-Won Joh, Kyung-Suk Suh

**Affiliations:** 1 Department of Internal Medicine and Liver Research Institute, Seoul National University College of Medicine, 101 Daehak-ro, Jongno-gu, Seoul 03080, Korea; 2 Institute for Digestive Research, Digestive Disease Center, Department of Internal Medicine, Soonchunhyang University College of Medicine, Seoul 04401, Korea; 3 Department of Internal Medicine, CHA Gangnam Medical Center, CHA University School of Medicine, Seoul 06135, Korea; 4 Department of Internal Medicine, Samsung Medical Center, Sungkyunkwan University School of Medicine, Seoul 06351, Korea; 5 Center for Liver Cancer, National Cancer Center, Goyang-Si, Gyeonggi-Do 10408, Korea; 6 Department of Surgery, Seoul National University College of Medicine, Seoul 03080, Korea; 7 Department of Surgery, Samsung Medical Center, Sungkyunkwan University School of Medicine, Seoul 06351, Korea

**Keywords:** hepatocellular carcinoma, living donor liver transplantation, MoRAL score, survival, validation

## Abstract

Background and Aims: Several models have been developed to predict tumor the recurrence of hepatocellular carcinoma (HCC) after liver transplantation besides the conventional Milan criteria (MC), including the MoRAL score. This study aimed to compare the prognostication power of the MoRAL score to most models designed so far in the Eastern and Western countries. Methods: This study included 564 patients who underwent living donor liver transplantation (LDLT) in three large-volume hospitals in Korea. The primary and secondary endpoints were time-to-recurrence, and overall survival (OS), respectively. The performance of the MoRAL score was compared with those of other various Liver transplantation (LT) criteria, including the Milan criteria, University of California San Francisco (UCSF) criteria, up-to-seven criteria, Kyoto criteria, AFP model, total tumor volume/AFP criteria, Metroticket 2.0 model, and Weill Cornell Medical College group model. Results: The median follow-up duration was 78.1 months. Among all models assessed, the MoRAL score showed the best discrimination function for predicting the risk of tumor recurrence after LT, with *c*-index of 0.78, compared to other models (all *p* < 0.001). The MoRAL score also represented the best calibration function by Hosmer-Lemeshow test (*p* = 0.15). Especially in the beyond-MC sub-cohort, the MoRAL score predicted tumor recurrence (*c*-index, 0.80) and overall survival (OS) (*c*-index, 0.70) significantly better than any other models (all *p* < 0.001). When the MoRAL score was low (<314.8), the five-year cumulative risks of tumor recurrence and death were excellent in beyond-MC (27.8%, and 20.5%, respectively) and within-MC (16.3%, and 21.1%, respectively) sub-cohorts. Conclusions: The MoRAL score provides the most refined prognostication for predicting HCC recurrence after LDLT.

## 1. Introduction 

Liver transplantation (LT) has been widely accepted as the treatment of choice for early-stage hepatocellular carcinoma (HCC) and end-stage liver disease [1,2]. Recently, LT eligibility criteria has been gradually expanded, especially for living donor LT (LDLT), due to excellent overall survival (OS) after LT [3]. Some pioneers in the field have suggested expanding the selection criteria for patients beyond the Milan criteria (MC), resulting in more patients being cured by LT at the expense of a higher rate of recurrence [4,5,6,7].

Because of the limited number of deceased donors, a patient beyond the MC was rarely transplanted with a liver from a deceased donor. However, LDLT provides an alternative to deceased donor LT (DDLT), allowing many centers to provide transplants to patients beyond the MC. In Asia, LDLT is more frequently performed than DDLT because there are fewer deceased donors due to different cultural and religious backgrounds from the West. The rapid expansion of LDLT criteria in Asia for patients beyond the MC [8,9,10,11] has demanded a model that can discriminate patients who can undergo transplant from those who should be excluded.

We previously published the results from the derivation of the model to predict tumor recurrence after LDLT for HCC beyond the MC (MoRAL) and its external validation [12]. To establish the model, a multicenter study was conducted, that involved consecutive patients who underwent LDLT for HCC. The MoRAL score ($11\;\times \;\sqrt{\mathrm{PIVKA}}+\;2\;\times \;\sqrt{\mathrm{AFP}}$), using serum alpha-fetoprotein (AFP) and protein induced by vitamin K absence-II (PIVKA-II) levels, could predict tumor recurrence after LDLT and identify patients with low recurrence and long-term OS potential. Beyond-MC patients, with a low-MoRAL score (≤314.8), had an approximate five-year recurrence-free survival rate of 70% and an OS rate of 80%.

Interestingly, another prognostic model with similar designation (MORAL) was more recently developed by a group from Weill Cornell Medical College (WCM). They proposed pre-, post-, and combo-MORAL scores to predict tumor recurrence after LT, using the neutrophil-lymphocyte ratio (NLR), AFP, and tumor size, all of which were superior to the MC at predicting recurrence for patients with HCC at a single center in United States [13].

In performing LDLT, the safety of living donors should be the primary concern. Therefore, it is necessary to develop a model for LDLT that can exclude those high-risk patients to reduce the risk of futile transplantation and can include low-risk patients even when they are beyond the MC. We have established the MoRAL score to discriminate those patients with low risk of HCC recurrence after LDLT [12]. In this study, we investigated the long-term prognosis of LDLT recipients by the MoRAL score with multicenter long-term follow-up data, and compared the prognostication power between the MoRAL score and other models, that have been designed so far in Western and Eastern worlds.

## 2. Methods

### 2.1. Study Population

This multicenter study recruited patients who underwent LDLT for HCC and prospectively registered to the LDLT databank in three highly experienced LT centers in Korea: Seoul National University Hospital (SNUH; Seoul, Korea; between September 2001 and January 2013), Samsung Medical Center (SMC; Seoul, Korea; between June 2003 and July 2013), and Korea National Cancer Center (NCC; Goyang-Si, Gyeonggi-Do, Korea; between June 2003 and July 2013).

Donor safety was the primary concern of LDLT, and comprehensive informed consent of both, recipient and donor, were obtained. All LDLTs were performed after extensive and thorough multidisciplinary reviews, including the transplantation center and the divisions of internal medicine, surgery, radiology, and psychiatry. The diagnosis of HCC was primarily determined by the histological findings of liver explants. However, in the case of non-viable tumors in liver explants, we diagnosed HCC according to the non-invasive diagnostic criteria from the American Association for the Study of Liver Diseases [14]. Patients were excluded from the study if they lacked critical clinical and laboratory data, including serum AFP and PIVKA-II, within 1 month before LDLT.

Among a total of 637 patients who underwent LDLT for HCC during the study period, 73 patients with no available critical data were excluded. Finally, 564 patients were enrolled in this study.

### 2.2. Selection Criteria for LDLT

Patients within the MC who had an available donor, and wanted to be transplanted, were subjected to LDLT. For the patients beyond the MC, trans-arterial chemoembolization or sorafenib was preferentially recommended, and after that LDLT was performed when they were refractory to those treatments and had a strong request for LT. The absolute contraindication was composed of extrahepatic metastasis, previous or concurrent other malignancy, severe cardiopulmonary disorders, and active infection. Portal vein invasion was not an absolute contraindication. We provided LDLT to patients with portal vein invasion who demonstrated progressive functional decline, had no other effective treatment modality, and had a strong demand for LDLT to increase the chance of survival [15].

All LDLT was performed after obtaining fully informed written consent of both recipient and donor, as well as approval from multidisciplinary discussion between internal medicine, surgery, radiology, and psychiatry. Extensive workups, including chest computed tomography (CT), ^18^F-FDG positron emission tomography (PET)/CT, bone scan, and upper and lower gastrointestinal endoscopy were performed to confirm the absence of extrahepatic metastasis or malignancies, other than HCC.

### 2.3. Comparison with Various LT Criteria

The performance of the MoRAL score on predicting HCC recurrence was compared with those of other various LT criteria, including the Milan criteria, University of California San Francisco (UCSF) criteria [16], up-to-seven criteria [17], Kyoto criteria [9], AFP model [18], UCSF or up-to-seven and AFP [19], total tumor volume/AFP criteria [20]. Metroticket 2.0 Model (revised up-to-seven criteria) [21], and a model recently suggested by the WCM group [13].

The Milan criteria was selected because it is still a current standard of care in LT. Among various alternative criteria other than the MC, externally validated criteria either, in the seminal publication or subsequent studies, were adopted for comparison [22]. In addition, the most recently suggested prognostic model by the WCM group was also compared. To avoid confusion, the MORAL score, developed by the WCM group, was designated as WCM model in this article.

### 2.4. Statistical Analysis

The primary endpoint was time-to-recurrence of HCC, and the secondary endpoint was OS. The index date was defined as the date of LDLT, and the cutoff date was 12 February 2018. The cumulative tumor recurrence rate and OS were estimated, using the Kaplan-Meier method, and compared by log-rank test.

The prognostication power of the MoRAL score, and other models was analyzed by focusing on both, discrimination and calibration performance. Discrimination performance refers to the degree to which the model differentiates between patients who are likely to experience a recurrence or die, and those who are not. It was analyzed by both graphical and numerical assessments: Receiver operating characteristics (ROC) curve for the graphical assessment, and area under the ROC curve (AUROC) or Harrell’s concordance index (*c*-index) for the numerical assessment. The DeLong method was used for statistical comparison of the ROC curves. The calibration performance, which provides indication accordance between estimated risks and observed outcomes, was evaluated with the Hosmer-Lemeshow goodness-of-fit test. Differences with a *P* value less than 0.05 were regarded as statistically significant.

All statistical analyses were performed using R version 3.4.3 (R Foundation for Statistical Computing, Vienna, Austria).

### 2.5. Ethical Considerations

This study followed the most recent ethical guidelines of the World Medical Association Declaration of Helsinki and was reviewed and approved by the Institutional Review Boards (IRB) of each participating center (SNUH IRB No. H-1208-063-421; SMC IRB No. 2014-01-038; NCC IRB No. NCCNCS13791). Written informed consent was waived because of the retrospective nature of the study and that there was no study-specific intervention beyond routine clinical care. Medical records of the included patients were anonymized and de-identified before analysis.

## 3. Results

### 3.1. Baseline Characteristics

Table 1 shows the baseline characteristics, that are based on pre-LT laboratory findings and radiologic imaging of the study population, which are classified by MoRAL scores as follows: the low-MoRAL group (*n* = 501) was defined as a score less than 314.8; the high-MoRAL group (*n* = 63) was defined as a score greater than 314.8; the mean MoRAL score was 94.1 in the low-MoRAL group and 794.8 in the high-MoRAL group and; the proportion of Child-Pugh class was similar in both groups. In contrast, significant differences between the two groups were observed in maximal tumor size, number of nodules, and portal vein invasion. The high-MoRAL group exhibited a larger size, higher numbers of tumor, with more frequent portal vein invasion, than the low-MoRAL group. In addition, the proportion of patients with the diffuse or infiltrative types of HCC was significantly higher in the high-MoRAL group. The median follow-up duration was 78.1 (interquartile range, 39.0–110.5) months.

We examined the association of HCC recurrence with the baseline variables, including the components of the MoRAL score in this multicenter long-term follow-up cohort. Serum $\sqrt{\mathrm{AFP}}$ and $\sqrt{\mathrm{PIVKA}-\mathrm{II}}$ were independently associated with HCC recurrence, while NLR was not significantly associated with HCC recurrence in multivariate analysis (Table 2). 

### 3.2. Impact of the MoRAL Score in HCC Recurrence and OS

**Figure 1:**
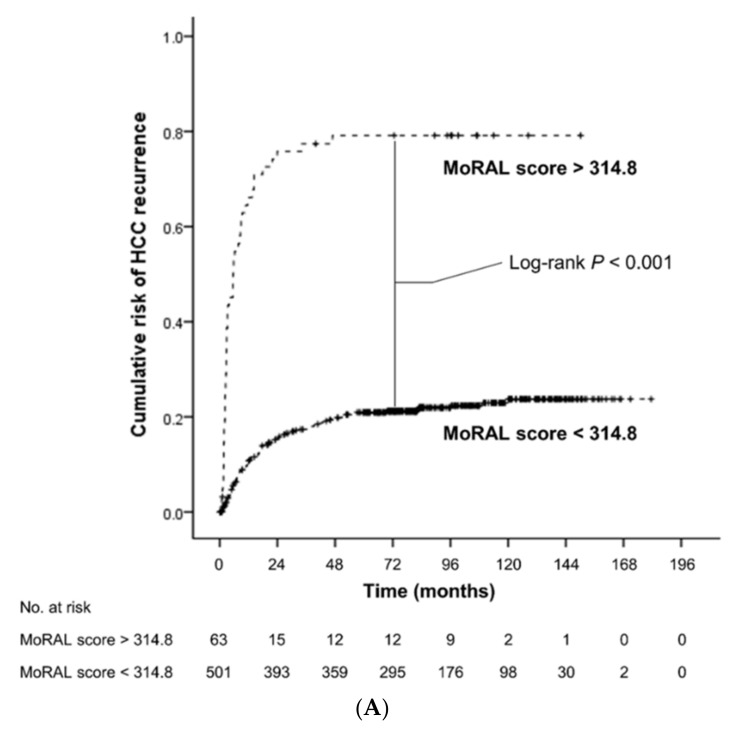

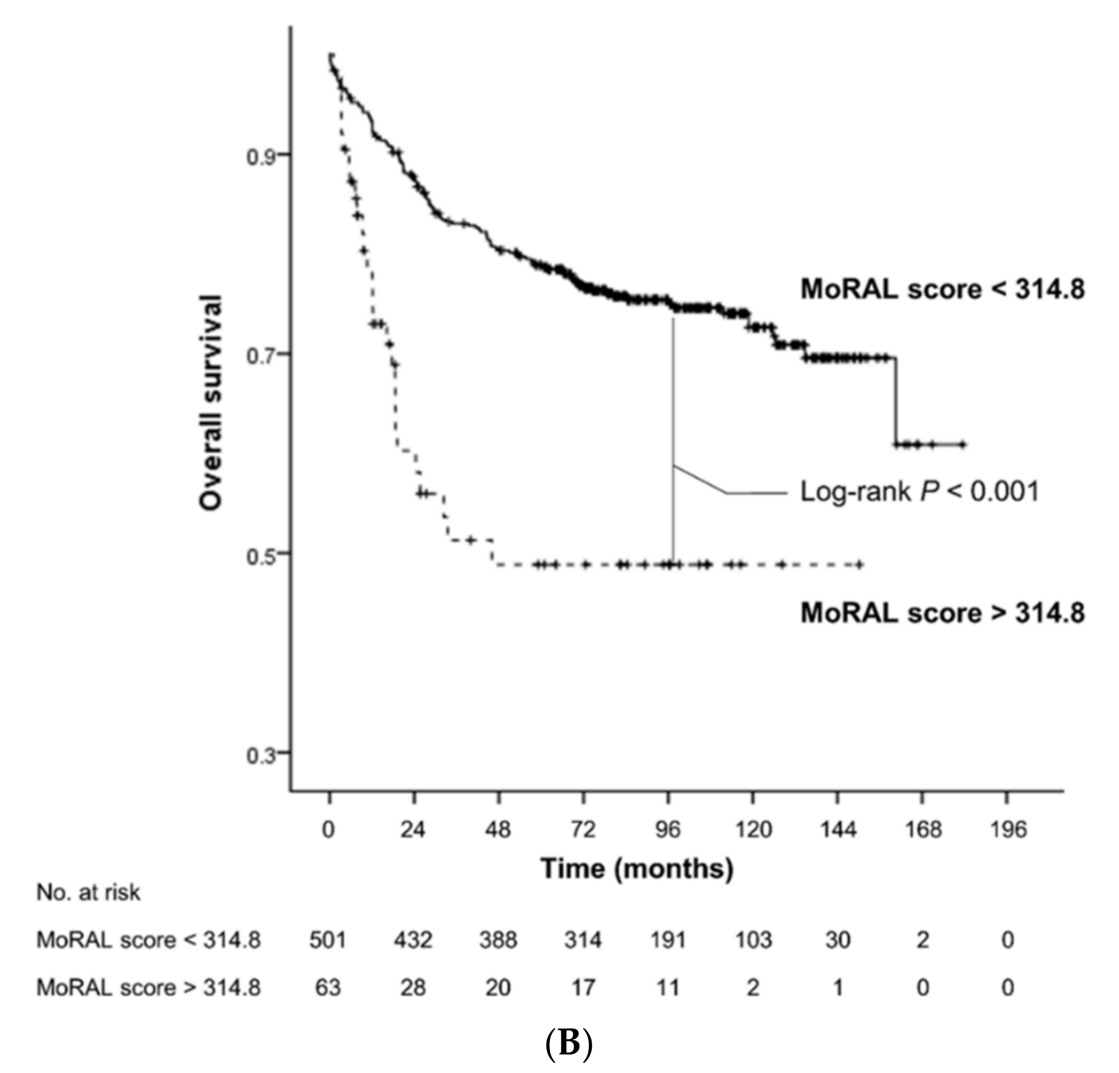
Kaplan-Meier estimates of cumulative risk of HCC recurrence (**A**) and overall survival (**B**) according to the MoRAL score. The low-MoRAL group (shown as a continuous line) was associated with significantly lower risk of HCC recurrence and overall death than the high-MoRAL group (shown as a dashed line).

The low-MoRAL group was associated with a significantly lower risk of tumor recurrence (hazard ratio [HR], 0.13; 95% confidence interval [CI], 0.09–0.19; *p* < 0.001; Figure 1A) and overall death (HR, 0.35; 95% CI, 0.23–0.54; *p* < 0.001; Figure 1B) than the high-MoRAL group. In the beyond-MC sub-cohort, patients with a low-MoRAL score had a significantly lower risk of tumor recurrence (HR, 0.17; 95% CI, 0.11–0.26; *p* < 0.001; Figure S1) and overall death (HR, 0.33; 95% CI, 0.19–0.55; *p* < 0.001; Figure S2). In the within-MC sub-cohort, although patients with a low-MoRAL score showed a significantly lower risk of tumor recurrence (HR, 0.27; 95% CI, 0.11–0.68; *p* = 0.005; Figure S3), while no significant difference in OS (HR, 0.57; 95% CI, 0.21–1.56; *p* = 0.28; Figure S4) was observed. Patients with a low-MoRAL score exhibited an excellent 5-year cumulative risk of tumor recurrence and death in the beyond-MC (27.8% and 20.5%, respectively), as well as in the within-MC sub-cohorts (16.3% and 21.1%, respectively). In contrast, patients with a high-MoRAL score showed a considerably higher risk of tumor recurrence and death (5-year rate of recurrence, 45.5%; death, 36.4%) than patients with a low-MoRAL score, even if they were within the MC.

### 3.3. Prognostication Power of the Models for Predicting HCC Recurrence and OS

The MoRAL score produced the best discrimination function for predicting tumor recurrence among all models, including current standard of care, MC. The *c*-indices were 0.77 (95% CI, 0.72–0.82) for the MoRAL score, 0.64 (95% CI, 0.60–0.67) for the MC. Although the WCM model showed relatively high *c*-index (0.69; 95% CI, 0.64–0.73) among the various LT criteria, the MoRAL score was superior to the WCM model. The MoRAL score showed a significantly higher *c*-index than any other LT criteria (all *p* < 0.001, Table 3A). In addition, the MoRAL score showed the best calibration function with a non-significant *p* value (*p* = 0.15) by the Hosmer-Lemeshow test; the WCM model and the AFP model, the second and third best models evaluated by *c*-index, were found to be less accurate with marginally significant *p* values (*p* = 0.07, and *p* = 0.06, respectively). These findings indicate that patients within a sub-group, that are classified by a MoRAL score, had a more homogeneous prognosis than patients within a subgroup classified by the others.

**Figure 2:**
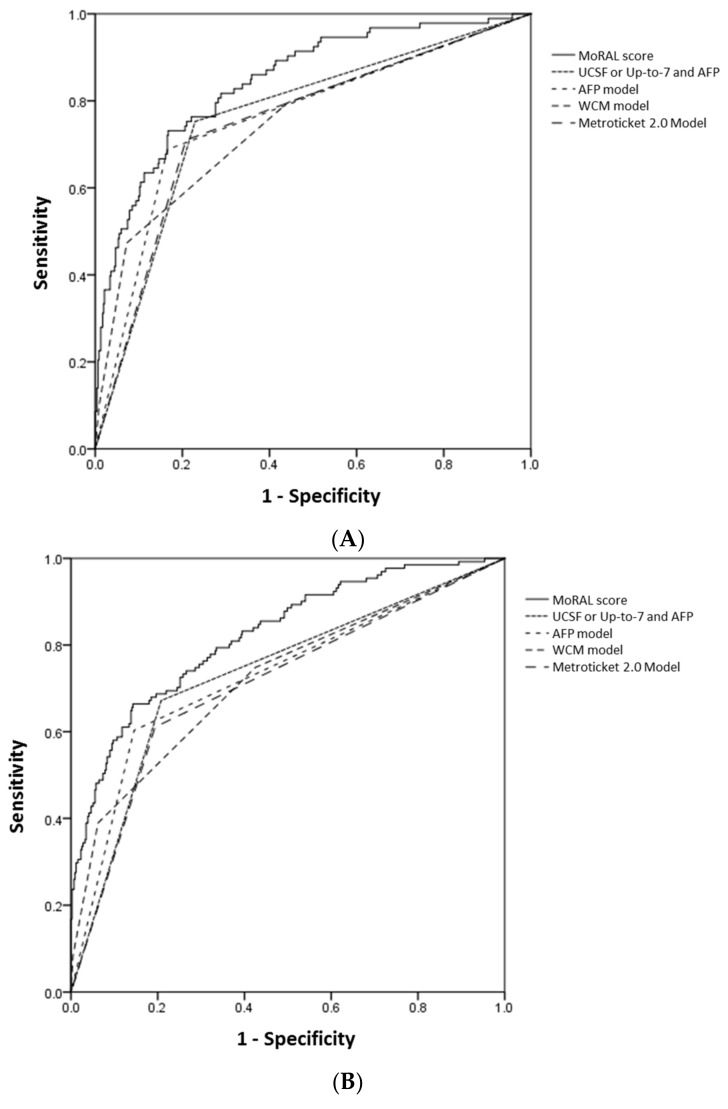

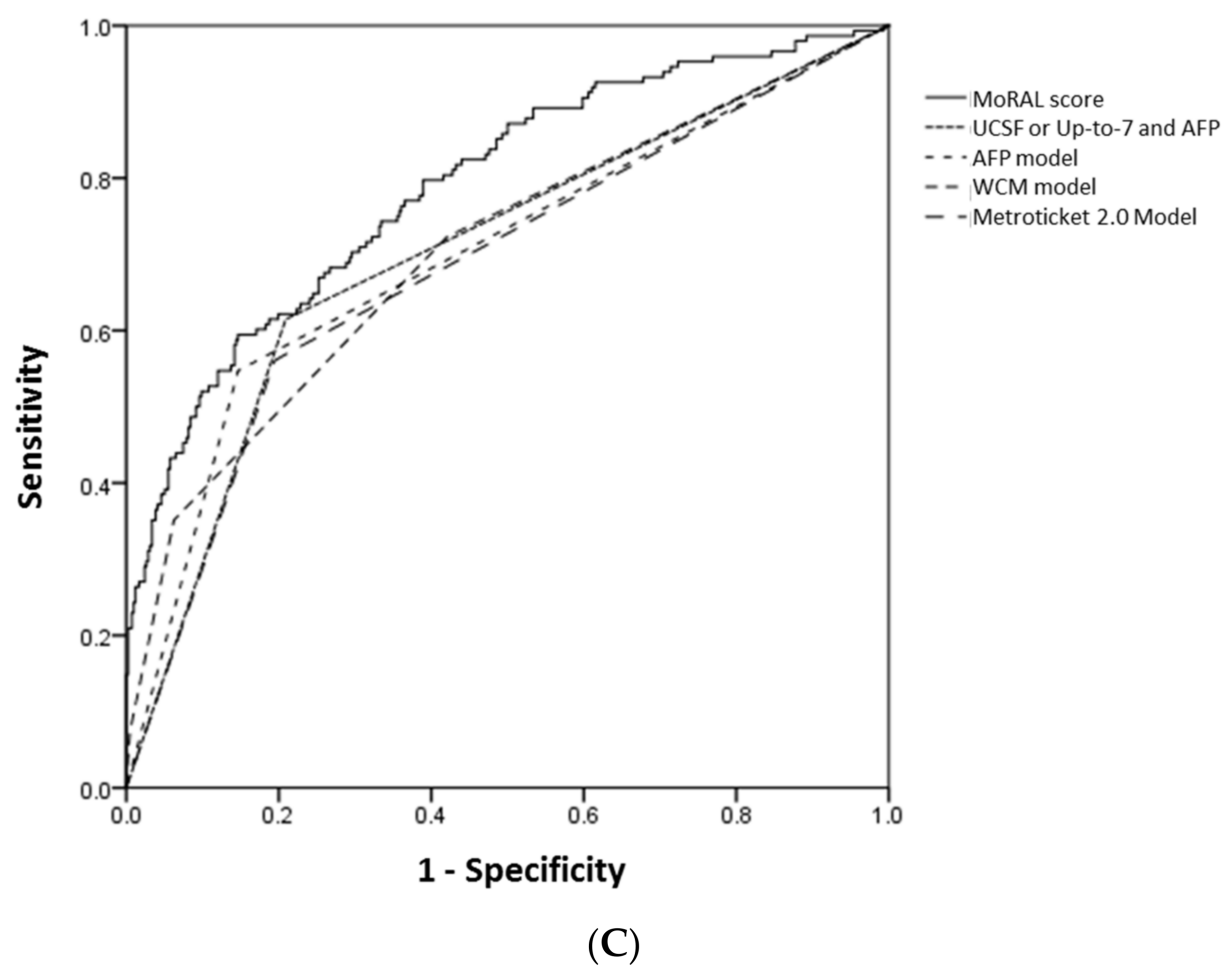
The ROC curves for 1-year (**A**), 3-year (**B**) and 5-year (**C**) HCC recurrence of the MoRAL score (shown as a continuous line) and the other models (shown as various dashed lines). The AUROCs of the MoRAL score for 1-year, 3-year, and 5-year recurrence risk were significantly higher than those of the other models.

For predicting tumor recurrence within five years, the MoRAL score consistently had better discrimination and calibration function than the other models. Among the whole study population, 93 of 446, 131 of 388, and 148 of 352 patients had recurred HCC within 1, 3, and 5 year, respectively. In the high-MoRAL group, 1-, 3-, and 5-year cumulative risks of tumor recurrence were as high as 66.1%, 77.4%, and 79.2%, meanwhile in the low-MoRAL group, 10.8%, 17.4%, and 21.0%, respectively. The ROC curves for 1-year, 3-year, and 5-year recurrence of the models are plotted in Figure 2. The AUROCs of the MoRAL score for 1-year, 3-year, and 5-year recurrence risk were significantly higher (1-year: 0.85; 3-year: 0.82; 5-year: 0.79) than the other models (all *p* < 0.001, Table 3B). The MoRAL score had good calibration function with non-significant *P* values by the Hosmer-Lemeshow test for 3-year and 5-year recurrence (both *p* = 0.10).

The prognostication power of the models was generally low for predicting the OS. Among the various prediction models, the MoRAL score showed the highest *c*-index (0.64; 95% CI, 0.59–0.69; Table 4).

### 3.4. Prognostication Power of the Models in the Beyond-MC and Within-MC Sub-Cohorts

Among the 564 patients included in this study, 205 patients were beyond the MC. In this beyond-MC sub-cohort, the MoRAL score showed a greater predictive power for tumor recurrence (*c*-index = 0.80) than that in the entire cohort (*c*-index = 0.77). In addition, the MoRAL score in this sub-cohort provided a significantly higher *c*-index (0.80; 95% CI, 0.73–0.86) than any other models (all *p* < 0.001, Table S1). In the within-MC sub-cohort (*n* = 359), the MoRAL score showed consistently good prognostication power for tumor recurrence. The *c*-index was significantly higher in the MoRAL score (0.69; 95% CI, 0.62–0.76) than by the other models (all *p* < 0.05, Table S1). 

The MoRAL score in the beyond-MC sub-cohort consistently demonstrated significantly better predictive power of tumor recurrence of less than 5 years, than any other model. The MoRAL score showed significantly higher AUROCs for 1-year, 3-year, and 5-year recurrence (1-year, 0.85; 3-year, 0.88; 5-year, 0.85) than any other model (all *p* < 0.001, Table S2). In the within-MC sub-cohort, the MoRAL score showed generally higher AUROCs for predicting 1-year, 3-year, and 5-year recurrence (1-year, 0.76; 3-year, 0.71; 5-year, 0.69) than the other models (Table S3).

For predicting OS, the MoRAL score held a more significantly improved *c*-index (0.70; 95% CI, 0.63–0.78) in the beyond-MC sub-cohort than in the entire cohort. The MoRAL score showed significantly higher *c*-index for predicting OS than the other models, except the AFP model (Table S4). In the within-MC sub-cohort, the predictive power of every model decreased with relatively low *c*-indices, and no significant statistical differences were found in the top four models, including the MoRAL score (Table S4).

## 4. Discussion

In this study, we found that the MoRAL score performed better in predicting the risk of tumor recurrence after LDLT among other currently available models, including the MC, current standard of care, and several other risk scores, which use AFP and tumor burden, including the WCM model [13], AFP model [18], UCSF or up-to-seven and AFP [19], and Metroticket 2.0 model [21]. The MoRAL score had the best calibration function by the Hosmer-Lemeshow test, which indicated that the patients within a subgroup classified by MoRAL score had the most homogeneous prognosis. Moreover, the performance of the MoRAL score for predicting tumor recurrence and OS in the beyond-MC sub-cohort was better than that of the WCM model, the most recent and the second most accurate model evaluated by *c*-index in this study. In the within-MC sub-cohort, the MoRAL score consistently showed better prognostication power for long- and short-term risk of tumor recurrence.

Basically, the expression of AFP in HCC tissues was shown to be related to the biological aggressiveness of HCC [22]. The serum level of AFP showed close correlation with tumor differentiation and aggressiveness [23], and was also a suggested indicator, reflecting tumor doubling time [24]. Accordingly, preoperative high AFP has been consistently mentioned as one of the risk factors associated with HCC recurrence after liver transplantation [25,26,27]. Although the favorable performance of PIVKA-II, another representative tumor marker of HCC, for diagnosis of HCC was demonstrated and widely used in Asian countries, the experiences in European countries, using PIVKA-II as a screening tool, have been relatively limited compared to Asian countries [28]. Recently, however, mounting evidence regarding the usefulness of PIVKA-II for HCC diagnosis, is accumulating [28,29,30]. PIVKA-II was also proven, to act not only a diagnostic biomarker, but as an independent prognostic factor in HCC patients [9]. Serum PIVKA-II was reported to be strongly associated with microvascular invasion and extrahepatic metastasis, even in early HCC [28,31]. These preceding studies support the clinical relevance of the MoRAL score, which consisted of AFP and PIVKA-II, in order to predict the prognosis of HCC patients after liver transplantation. We have developed the prediction model using most well validated and widely used two tumor markers in real clinical practice.

Worldwide, HCC is the second leading cause of cancer-related mortality [3], and its incidence has increased dramatically in the last few decades [32]. Less than one-third of patients with HCC are diagnosed at an early stage, when curative treatment is possible [33]. For HCC patients who are unsuitable for resection, LT is the optimal treatment modality for a cure. Therefore, it is necessary to identify a method for discriminating eligible patients who are at a low risk of tumor recurrence, even when beyond the MC. 

In our previous study [12], we established the MoRAL score, which is a simple and objective method of using serum tumor markers, AFP and PIVKA-II. With the MoRAL score, a clinician could identify highly selected patients beyond the MC, who are at a low risk of tumor recurrence after LDLT. More recently, another prognostic model was developed and published by Weill Cornell Medical College group in 2017, with similar designation of the MoRAL score. We named the new score as WCM model in our study to prevent confusion in comparison. The WCM model was composed of AFP, NLR, and tumor size. In the derivation cohort of our previous study, however, the maximal tumor size had been significantly associated with serum AFP, and the number and type of tumor with serum PIVKA-II, although those tumor factors had been associated with HCC recurrence. Moreover, it is difficult to measure the exact size and number of tumors in patients with infiltrative type of HCC. We finally developed a prediction model, with objective and reproducible variables; AFP and PIVKA-II. In the present study, we also examined the association of the NLR and HCC recurrence to find that there was no significant association by both univariate and multivariate analysis. The NLR is a marker, that has been originally suggested as a prognostic factor, which reflects an inflammatory response to tumor in patients with hepatic resection [34]. For the recipients of LT, however, its role tends to be diluted as all the recipients should get immunosuppression with various agents. 

In this long-term multicenter follow-up study, we found that a low-MoRAL score (<314.8) was associated with a significantly lower risk of tumor recurrence and overall death than the high-MoRAL score (>314.8). In the beyond-MC sub-cohort, the patients with a low-MoRAL score experienced a significantly lower risk of tumor recurrence and overall death than those with a high-MoRAL score. The five-year tumor recurrence rate of patients beyond the MC, with a low-MoRAL score, was only 27.8%, which is comparable with the scores of patients within the MC. If the MoRAL score is low, the risks of tumor recurrence and death, beyond the MC patients, are comparable to those of within the MC patients. If the MoRAL score is high, about a half of the patients may experience tumor recurrence, even if they are within the MC. In Asian countries, where LDLT is rapidly expanding [35], the MoRAL score may provide refined prognostication to help make decisions with patients and donors.

The MoRAL score showed the best prognostication power for tumor recurrence after LDLT (*c*-index, 0.77) among all LT criterion tested. Especially in predicting short-term tumor recurrence, the MoRAL score consistently had the best discrimination function. Early recurrence of HCC is known to shorten survival after LT [36], which implies that these tumors are biologically more aggressive [37,38,39]. In this study, the MoRAL score clearly distinguished the patients with poor prognosis after LT, due to the high likelihood of tumor recurrence. In particular, in the beyond-MC sub-cohort, the MoRAL score showed greater predictive power for tumor recurrence than in the original cohort. The performance of the MoRAL score was maximized when predicting tumor recurrence in HCC patients beyond the MC. This was basically because the MoRAL score had originated from Cox proportional hazard analyses for tumor recurrence, after LDLT, in beyond the MC patients. Although, the recurrence of HCC, after LT, is associated with poor overall survival, the prediction of recurrence is not directly linked to that of overall survival because other factors, including post-recurrence treatment, have a significant impact on overall survival. Recently, many studies have shown the favorable impact of treatment, including surgery after post-LC recurrence [40,41,42]. Therefore, the MoRAL score may better predict HCC recurrence among patients with HCC beyond the MC, although we also found that the MoRAL score could predict overall survival, as well as recurrence-free survival in both, beyond, and within the MC cohort.

In real clinical practice, it is rare for anyone outside of the MC to receive a transplant from a decent deceased donor, due to the scarcity of deceased donors [6]. Because of this rarity and the small number of LDLTs completed in the West [43], these findings on the MoRAL score may have more implications in Asian countries than in the West. The performance of the MoRAL score for predicting OS, as well as HCC recurrence, was the most significant especially in the beyond-MC sub-cohort. In the within-MC sub-cohort, the prognostication power of the MoRAL score was lower than in the beyond-MC sub-cohort. Since patients in the within-MC were clearly eligible for LT, the need for risk-predicting model is relatively small, and thus the usefulness of the MoRAL score is maximized in the beyond-MC patients, which requires discrimination.

Our study has several limitations. First, this cohort study included data from three highly experienced, large-volume LT centers. Moreover, this study represents the regional experiences of South Korea, where 70–80% of HCC patients have hepatitis B virus infection [44]. Therefore, our results may lack generalizability because most LTs outside of Asia are performed for HCC patients, with different underlying etiologies, such as non-alcoholic fatty liver disease or HCV. Second, only patients who underwent LDLT—not DDLT—were included in this study. Therefore, it is uncertain whether the MoRAL score could be applied for patients who undergo DDLT. Further prospective studies are necessary to evaluate whether the MoRAL score can be applied to those patients. There are several additional limitations to discuss, mainly related to the retrospective nature of this study. Among the study population, within the MC patients were relatively homogeneous; while beyond the MC patients had to be quite heterogeneous. The heterogeneity was inevitable in order to include all the patients who underwent LDLT to reflect real-world practice as much as possible. Nevertheless, the MoRAL score discriminated HCC recurrence well among the highly heterogeneous beyond-MC group making a good distinction. Moreover, MoRAL showed good calibration and discriminant functions in this study, which suggests that patients within the high-MoRAL score have a relatively similar prognosis, but have a significantly different prognosis from patients in the low-MoRAL group. Another limitation is that the number of patients with high-MoRAL score is small compared to those with low-MoRAL score. Since the MoRAL score was developed to discriminate HCC patients with a high risk of recurrence after LDLT, the cut-off value was set to the highest 25th percentile value in the derivation set of the original study subjects. Accordingly, the number of patients with high MoRAL score is bound to be small (high-MoRAL:low-MoRAL = 1:3 at index date). Despite the limited number of patients with high MoRAL score, the differences in cumulative risk of HCC recurrence and overall survival were evident and statistically significant.

## 5. Conclusions

In conclusion, the MoRAL score showed the best performance for predicting the risk of tumor recurrence after LT among all models studied, including the MC, a current standard of care, and various other externally validated LT criteria. The MoRAL score represents a favorable calibration function, indicating that patients within a subgroup classified by MoRAL score had a homogeneous prognosis. The MoRAL score provides the most refined prognostication for predicting HCC recurrence after LDLT.

## Figures and Tables

**Table 1:** Baseline characteristics according to the MoRAL score.

Characteristics	MoRAL < 314.8 (*n* = 501)	MoRAL > 314.8 (*n* = 63)	*p* Value
Age, year	49.1 ± 19.1	55.1 ± 9.3	<0.001
Male, *N* (%)	409 (81.6%)	56 (88.9%)	0.10
AFP, ng/mL	13 (5.9, 58.1)	810 (126.6, 6509.4)	<0.001
PIVKA-II, AU/mL	26 (15, 61)	1200 (726.5, 2218.5)	<0.001
MoRAL score	66.7 (51.87, 113.79)	478.7 (386.53, 694.92)	<0.001
Child-Pugh class: A/B/C, *N* (%)	299/119/83 (59.7/23.8/16.5%)	36/16/11 (57.7/25/17.3%)	0.66
BCLC stage: 0/A/B/C/D, *N* (%)	61/226/81/36/97 (12.2/45.1/16.2/7.2/19.4%)	1/9/18/23/12 (1.6/14.3/28.6/36.5/19%)	<0.001
Maximal size of HCC, cm	2.5 ± 1.5	6.0 ± 3.2	<0.001
Number of HCC, *N*	2.3 ± 2.7	5.9 ± 8.4	<0.001
Type of HCC: nodular/diffuse or infiltrative, *N* (%)	475/26 (94.9/5.1%)	35/28 (54.9/45.1%)	<0.001
Portal vein invasion, *N* (%)	54 (10.7%)	29 (46%)	<0.001

Data were presented as mean ± SD or median (IQR). AFP, alpha-fetoprotein; PIVKA-II, protein induced by vitamin K absence-II; BCLC, Barcelona Clinic Liver Cancer; HCC, hepatocellular carcinoma; SD, standard deviation; IQR, interquartile range.

**Table 2:** Univariate and multivariate analysis of variables associated with hepatocellular carcinoma (HCC) recurrence.

Variables	Univariate Analysis		Multivariate Analysis	
	HR (95% CI)	*p* Value	HR (95% CI)	*p* Value
Serum $\sqrt{\mathrm{AFP}}$	1.003 (1.003–1.004)	<0.001	1.002 (1.001–1.003)	<0.001
Serum $\sqrt{\mathrm{PIVKA}-\mathrm{II}}$	1.02 (1.016–1.023)	<0.001	1.018 (1.015–1.022)	<0.001
NLR	1.01 (0.99–1.04)	0.32	1.003 (0.97–1.03)	0.87
Maximal size of HCC	1.34 (1.27–1.40)	<0.001		
Number of HCC	1.08 (1.05–1.10)	<0.001		
Diffuse or infiltrative type of HCC	3.27 (2.33–4.58)	<0.001		

HCC, hepatocellular carcinoma; HR, hazard ratio; AFP, alpha-fetoprotein; PIVKA-II, protein induced by vitamin K absence-II; NLR; neutrophil-to-lymphocyte ratio.

**Table 3:** Comparison of discrimination function for predicting hepatocellular carcinoma recurrence by various models in the entire cohort.

**A: *C*-Indices for Predicting HCC Recurrence by Various Models in the Entire Cohort**										
**Models**		***C*-Index**			**95% Confidence Interval**					*p* Value *
					**Lower**			**Upper**		
MoRAL score		0.77			0.73			0.82		
WCM model		0.69			0.65			0.73		<0.001
AFP model		0.68			0.65			0.72		<0.001
UCSF or Up-to-seven and AFP		0.68			0.64			0.71		<0.001
Metroticket 2.0 Model		0.68			0.64			0.71		<0.001
Milan criteria		0.64			0.60			0.67		<0.001
UCSF criteria		0.62			0.58			0.65		<0.001
Up-to-seven criteria		0.61			0.58			0.64		<0.001
Total tumor volume/AFP criteria		0.52			0.49			0.55		<0.001
Kyoto criteria		0.50			0.47			0.53		<0.001
**B: Comparison of the AUROC for Predicting 1-Year, 3-Year, and 5-Year HCC Recurrence by Various Predicting Models in the Entire Cohort**										
**Models**			**AUROC**	**95% CI**			*p* Value *		***p* Value ^†^**	
				**Lower**		**Upper**				
1 year	MoRAL score		0.85	0.80		0.89	<0.001			
	WCM model		0.75	0.70		0.81	<0.001		<0.001	
	AFP model		0.76	0.70		0.82	<0.001		<0.001	
	UCSF or Up-to-seven and AFP		0.76	0.71		0.82	<0.001		<0.001	
	Metroticket 2.0 Model		0.75	0.69		0.81	<0.001		<0.001	
3 year	MoRAL score		0.82	0.78		0.87	<0.001			
	WCM model		0.72	0.67		0.78	<0.001		<0.001	
	AFP model		0.73	0.68		0.78	<0.001		<0.001	
	UCSF or Up-to-seven and AFP		0.73	0.68		0.78	<0.001		<0.001	
	Metroticket 2.0 Model		0.71	0.65		0.76	<0.001		<0.001	
5 year	MoRAL score		0.79	0.75		0.83	<0.001			
	WCM model		0.70	0.65		0.76	<0.001		<0.001	
	AFP model		0.70	0.65		0.75	<0.001		<0.001	
	UCSF or Up-to-seven and AFP		0.70	0.65		0.75	<0.001		<0.001	
	Metroticket 2.0 Model		0.68	0.63		0.74	<0.001		<0.001	

(**A**) * Compare to the *c*-index of the MoRAL score. HCC, hepatocellular carcinoma; WCM, Weill Cornell Medical College; AFP, alpha-fetoprotein; UCSF, University of California San Francisco. (**B**) * Null hypothesis: Actual area = 0.5, ^†^ Compare to the AUROC of the MoRAL score. AUROC, area under the receiver operating characteristics curve; HCC, hepatocellular carcinoma; CI, confidence interval; WCM, Weill Cornell Medical College; AFP, alpha-fetoprotein, UCSF, University of California San Francisco.

**Table 4:** *C*-indices for predicting overall survival by various models.

Models	*C*-Index	95% Confidence Interval		*p* Value *
		Lower	Upper	
MoRAL score	0.64	0.59	0.69	
WCM model	0.62	0.58	0.66	0.16
AFP model	0.58	0.55	0.62	0.004
UCSF or Up-to-seven and AFP	0.58	0.54	0.62	0.003
Metroticket 2.0 Model	0.58	0.54	0.61	0.002
UCSF criteria	0.55	0.51	0.58	<0.001
Milan criteria	0.54	0.50	0.58	<0.001
Up-to-seven criteria	0.53	0.50	0.56	<0.001
Total tumor volume/AFP criteria	0.52	0.49	0.55	<0.001
Kyoto criteria	0.51	0.48	0.54	<0.001

* Compare to the *c*-index of the MoRAL score. WCM, Weill Cornell Medical College; AFP, alpha-fetoprotein; UCSF, University of California San Francisco.

## Supplementary Materials

The following are available online at https://www.mdpi.com/2072-6694/11/9/1295/s1, Table S1: C-indices for predicting HCC recurrence by various models in the beyond-MC and within-MC subcohorts, Table S2: Comparison of the area under the ROC curve for predicting 1-year, 3-year, and 5-year HCC recurrence by various predicting models in the beyond-MC subcohort, Table S3: Comparison of the area under the ROC curve for predicting 1-year, 3-year, and 5-year HCC recurrence various predicting models in the within-MC subcohort, Table S4: C-indices for predicting overall survival by various models in the beyond-MC and within-MC subcohort, Figure S1: Kaplan-Meier estimates of cumulative risk of HCC recurrence according to the MoRAL score in the beyond-MC subcohort, Figure S2: Kaplan-Meier estimates of overall survival according to the MoRAL score in the beyond-MC subcohort, Figure S3: Kaplan-Meier estimates of cumulative risk of HCC recurrence according to the MoRAL score in the within-MC subcohort, Figure S4: Kaplan-Meier estimates of overall survival according to the MoRAL score in the within-MC subcohort.
