# Supplementary material for: Comparison of Models for Tumor Recurrence after Liver Transplantation for the Patients with Hepatocellular Carcinoma: A Multicenter Long-Term Follow-Up Study
Source: Cancers (Basel). 2019 Sep 3;11(9):1295. doi: 10.3390/cancers11091295 (PMC6769632; doi:10.3390/cancers11091295)
Supplement: Supplementary file 1 [file cancers-11-01295-s001.pdf]

Supplementary Materials: Comparison of Models for Tumor Recurrence after Liver Transplantation for the Patients with Hepatocellular Carcinoma: A Multicenter Long-Term Follow-Up Study

Young Chang, Yuri Cho, Jeong-Hoon Lee, Yun Bin Lee, Eun Ju Cho, Su Jong Yu, Dong Hyun Sinn, Bo Hyun Kim, Seoung Hoon Kim, Nam-Joon Yi, Kwang-Woong Lee, Jong Man Kim, Joong-Won Park, Yoon Jun Kim, Jung-Hwan Yoon, Jae-Won Joh and Kyung-Suk Suh

Table S1. *C*-indices for predicting HCC recurrence by various models in the beyond-MC and within-MC subcohorts.

		***C*-Index**	**95% CI**		***p* Value ***
			**Lower**	**Upper**	
Beyond-MC subcohort	MoRAL score	0.80	0.73	0.86	
	WCM model	0.70	0.64	0.75	<0.001
	AFP model	0.65	0.60	0.70	<0.001
	UCSF or Up-to-seven and AFP	0.67	0.61	0.72	<0.001
	Metroticket 2.0 Model	0.61	0.56	0.66	<0.001
	UCSF criteria	0.53	0.48	0.59	<0.001
	Up-to-seven criteria	0.55	0.51	0.60	<0.001
	Total tumor volume/AFP criteria	0.61	0.55	0.66	<0.001
	Kyoto criteria	0.61	0.56	0.66	<0.001
Within-MC subcohort	MoRAL score	0.69	0.62	0.76	
	WCM model	0.61	0.55	0.67	0.03
	AFP model	0.59	0.56	0.62	0.002
	UCSF or Up-to-seven and AFP	0.62	0.57	0.66	0.02
	Metroticket 2.0 Model	0.58	0.55	0.61	<0.001
	UCSF criteria	0.52	0.51	0.53	<0.001
	Up-to-seven criteria	0.50	0.50	0.50	<0.001
	Total tumor volume/AFP criteria	0.57	0.54	0.61	<0.001
	Kyoto criteria	0.50	0.50	0.50	<0.001

* Compare to the *c*-index of the MoRAL score. HCC, hepatocellular carcinoma; MC, Milan criteria; CI, confidence interval; WCM, Weill Cornell Medical College; AFP, alpha-fetoprotein, UCSF, University of California San Francisco.

Table S2. Comparison of the area under the ROC curve for predicting 1-year, 3-year, and 5-year HCC recurrence by various predicting models in the beyond-MC subcohort.

		AUROC	95% CI		*p* Value *	*p* Value †
			Lower	Upper		
1 year	MoRAL score	0.85	0.80	0.90	<0.001	
	WCM model	0.74	0.66	0.82	<0.001	< 0.001
	AFP model	0.71	0.63	0.78	<0.001	< 0.001
	UCSF or Up-to-seven and AFP	0.71	0.64	0.79	<0.001	< 0.001
	Metroticket 2.0 Model	0.65	0.57	0.72	0.001	< 0.001
3 year	MoRAL score	0.88	0.84	0.93	<0.001	
	WCM model	0.74	0.67	0.81	<0.001	< 0.001
	AFP model	0.71	0.64	0.78	<0.001	< 0.001
	UCSF or Up-to-seven and AFP	0.73	0.66	0.80	<0.001	< 0.001
	Metroticket 2.0 Model	0.65	0.57	0.72	<0.001	< 0.001
5 year	MoRAL score	0.85	0.80	0.91	<0.001	
	WCM model	0.74	0.67	0.81	<0.001	< 0.001
	AFP model	0.69	0.61	0.76	<0.001	< 0.001
	UCSF or Up-to-seven and AFP	0.71	0.63	0.78	<0.001	< 0.001
	Metroticket 2.0 Model	0.63	0.55	0.71	0.001	< 0.001

* Null hypothesis: Actual area = 0.5. † Compare to the AUROC of the MoRAL score. AUROC, area under the receiver operating characteristics curve; HCC, hepatocellular carcinoma; MC, Milan criteria; CI, confidence interval; WCM, Weill Cornell Medical College; AFP, alpha-fetoprotein, UCSF, University of California San Francisco

Table S3. Comparison of the area under the ROC curve for predicting 1-year, 3-year, and 5-year HCC recurrence various predicting models in the within-MC subcohort.

		AUROC	95% CI		*p* Value *	*p* Value †
			Lower	Upper		
1 year	MoRAL score	0.76	0.66	0.87	<0.001	
	WCM model	0.68	0.57	0.79	0.002	0.12
	AFP model	0.65	0.52	0.77	0.009	0.02
	UCSF or Up-to-seven and AFP	0.70	0.59	0.81	<0.001	0.26
	Metroticket 2.0 Model	0.65	0.53	0.77	0.009	0.02
3 year	MoRAL score	0.71	0.63	0.79	<0.001	
	WCM model	0.63	0.54	0.72	0.003	0.06
	AFP model	0.61	0.51	0.70	0.02	0.005
	UCSF or Up-to-seven and AFP	0.64	0.55	0.73	0.002	0.08
	Metroticket 2.0 Model	0.58	0.49	0.68	0.06	<0.001
5 year	MoRAL score	0.69	0.62	0.76	<0.001	
	WCM model	0.60	0.52	0.68	0.01	0.04
	AFP model	0.59	0.51	0.67	0.03	0.004
	UCSF or Up-to-seven and AFP	0.61	0.53	0.70	0.005	0.046
	Metroticket 2.0 Model	0.57	0.49	0.66	0.08	<0.001

* Null hypothesis: Actual area = 0.5. † Compare to the AUROC of the MoRAL score. AUROC, area under the receiver operating characteristics curve; HCC, hepatocellular carcinoma; MC, Milan criteria; CI, confidence interval; WCM, Weill Cornell Medical College; AFP, alpha-fetoprotein, UCSF, University of California San Francisco.

Table S4. *C*-indices for predicting overall survival by various models in the beyond-MC and within-MC subcohort.

		C-index	95% CI		*p* Value *
			Lower	Upper	
Beyond-MC subcohort	MoRAL score	0.70	0.63	0.78	
	WCM model	0.63	0.56	0.70	0.01
	AFP model	0.58	0.51	0.64	<0.001
	UCSF or Up-to-seven and AFP	0.64	0.58	0.71	0.07
	Metroticket 2.0 Model	0.56	0.50	0.62	<0.001
	UCSF criteria	0.55	0.49	0.62	<0.001
	Up-to-seven criteria	0.52	0.47	0.58	<0.001
	Total tumor volume/AFP criteria	0.56	0.49	0.62	<0.001
	Kyoto criteria	0.57	0.51	0.64	<0.001
Within-MC subcohort	MoRAL score	0.59	0.53	0.66	
	WCM model	0.61	0.55	0.66	0.63
	AFP model	0.56	0.53	0.58	0.11
	UCSF or Up-to-seven and AFP	0.57	0.53	0.61	0.19
	Metroticket 2.0 Model	0.54	0.52	0.57	0.04
	UCSF criteria	0.51	0.50	0.51	<0.001
	Up-to-seven criteria	0.50	0.50	0.50	<0.001
	Total tumor volume/AFP criteria	0.55	0.52	0.58	0.07
	Kyoto criteria	0.50	0.50	0.50	<0.001

* Compare to the *c*-index of the MoRAL score. MC, Milan criteria; CI, confidence interval; WCM, Weill Cornell Medical College; AFP, alpha-fetoprotein; UCSF, University of California San Francisco.

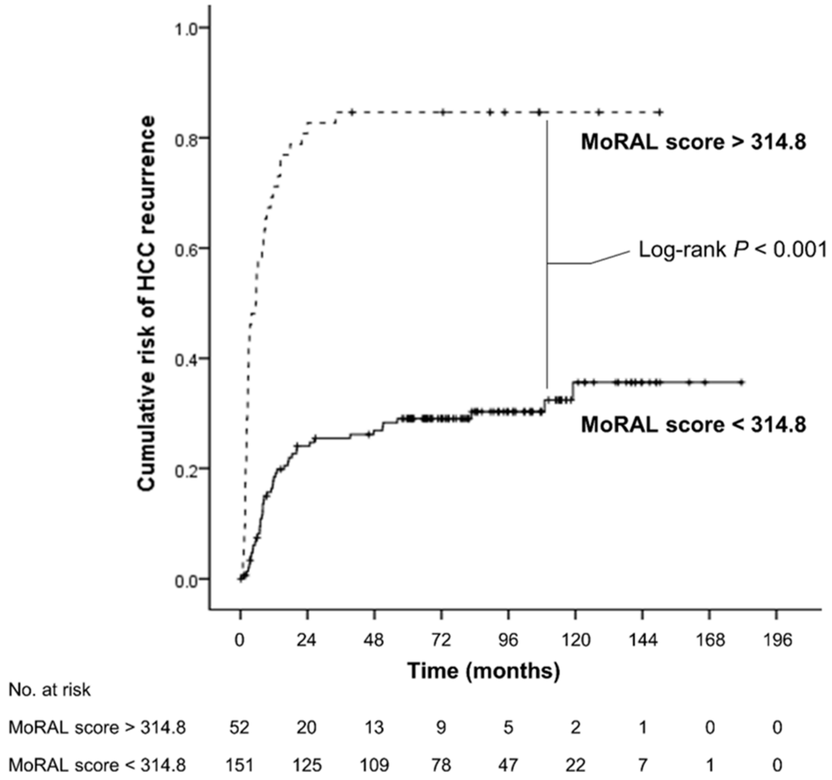

Figure S1. Kaplan-Meier estimates of cumulative risk of HCC recurrence according to the MoRAL score in the beyond-MC subcohort. The low-MoRAL group (shown as a continuous line) was associated with significantly lower risk of HCC recurrence than the high-MoRAL group (shown as a dashed line).

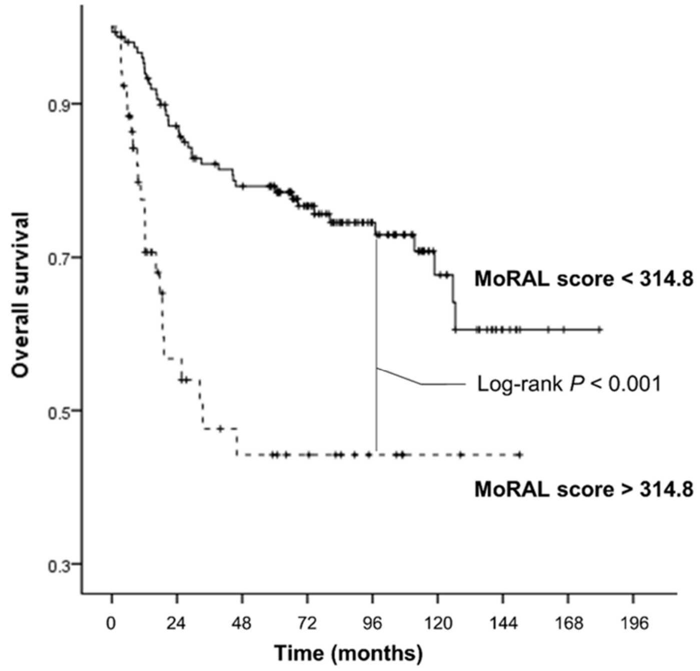

No. at risk									
MoRAL score < 314.8	151	108	102	73	44	20	7	1	0
MoRAL score > 314.8	52	9	7	7	4	2	1	0	0

Figure S2. Kaplan-Meier estimates of overall survival according to the MoRAL score in the beyond-MC subcohort. The low-MoRAL group (shown as a continuous line) was associated with significantly lower risk of overall death than the high-MoRAL group (shown as a dashed line).

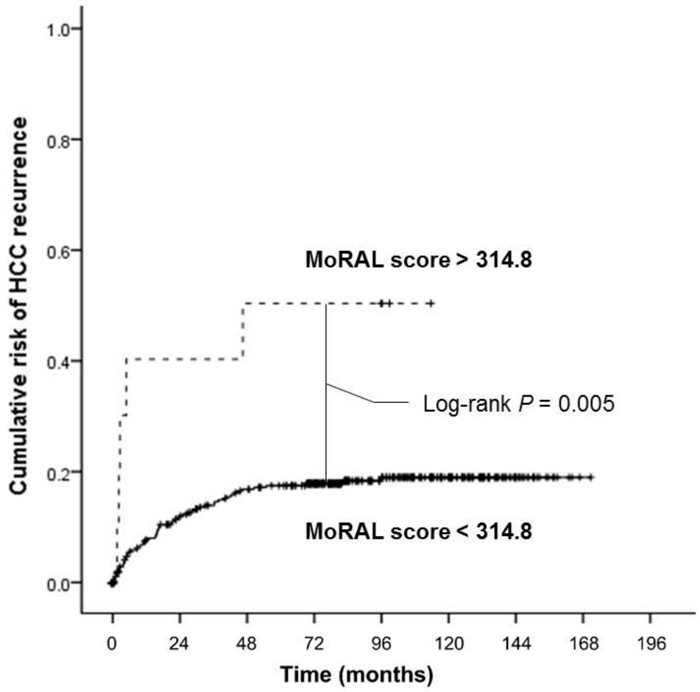

No. at risk									
MoRAL score > 314.8	51	9	7	7	4	2	1	0	0
MoRAL score < 314.8	349	285	257	222	134	78	23	1	0

Figure S3. Kaplan-Meier estimates of cumulative risk of HCC recurrence according to the MoRAL score in the within-MC subcohort. The low-MoRAL group (shown as a continuous line) was associated with significantly lower risk of HCC recurrence than the high-MoRAL group (shown as a dashed line).

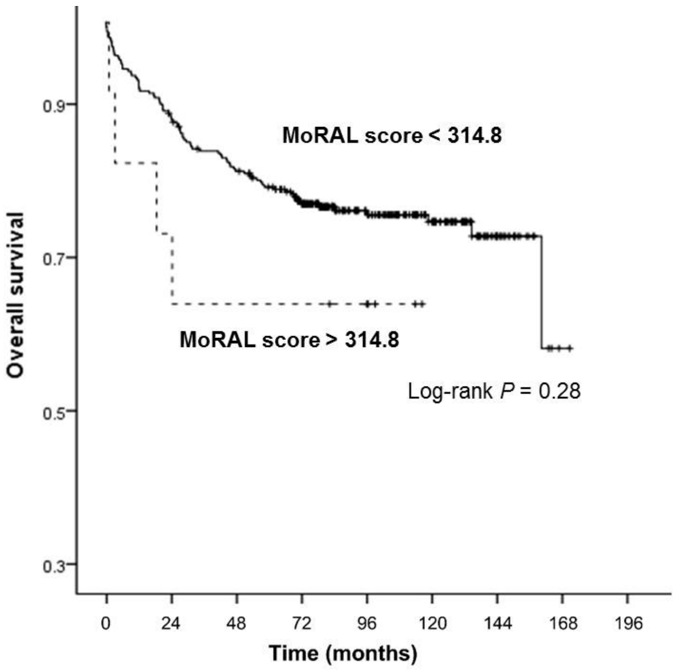

No. at risk

MoRAL score < 314.8	349	307	279	236	139	81	23	1	0
MoRAL score > 314.8	10	8	7	7	6	0	0	0	0

Figure S4. Kaplan-Meier estimates of overall survival according to the MoRAL score in the within-MC subcohort. The low-MoRAL group (shown as a continuous line) was associated with significantly lower risk of overall death than the high-MoRAL group (shown as a dashed line).

© 2019 by the authors. Licensee MDPI, Basel, Switzerland. This article is an open access article distributed under the terms and conditions of the Creative Commons Attribution (CC BY) license (http://creativecommons.org/licenses/by/4.0/).
